# Integrated visual transformer and flash attention for lip-to-speech generation GAN

**DOI:** 10.1038/s41598-024-55248-6

**Published:** 2024-02-24

**Authors:** Qiong Yang, Yuxuan Bai, Feng Liu, Wei Zhang

**Affiliations:** 1 School of Computer Science, Xi’an Polytechnic University, Xi’an, 710048 Shaanxi China; 2 Shaanxi Key Laboratory of Clothing Intelligence, School of Computer Science, Xi’an Polytechnic University, Xi’an, 710048 Shaanxi China; 3 China Mobile System Integration Co, Ltd, Xi’an, 710077 China

**Keywords:** Computer science, Information technology

## Abstract

Lip-to-Speech (LTS) generation is an emerging technology that is highly visible, widely supported, and rapidly evolving. LTS has a wide range of promising applications, including assisting speech impairment and improving speech interaction in virtual assistants and robots. However, the technique faces the following challenges: (1) Chinese lip-to-speech generation is poorly recognized. (2) The wide range of variation in lip-speaking is poorly aligned with lip movements. Addressing these challenges will contribute to advancing Lip-to-Speech (LTS) technology, enhancing the communication abilities, and improving the quality of life for individuals with disabilities. Currently, lip-to-speech generation techniques usually employ the GAN architecture but suffer from the following problems: The primary issue lies in the insufficient joint modeling of local and global lip movements, resulting in visual ambiguities and inadequate image representations. To solve these problems, we design Flash Attention GAN (FA-GAN) with the following features: (1) Vision and audio are separately coded, and lip motion is jointly modelled to improve speech recognition accuracy. (2) A multilevel Swin-transformer is introduced to improve image representation. (3) A hierarchical iterative generator is introduced to improve speech generation. (4) A flash attention mechanism is introduced to improve computational efficiency. Many experiments have indicated that FA-GAN can recognize Chinese and English datasets better than existing architectures, especially the recognition error rate of Chinese, which is only 43.19%, the lowest among the same type.

## Introduction

*Lip-to-speech*^1,4^* generation* is a highly regarded emerging technology that is experiencing rapid growth and many important breakthroughs. This technology not only provides a new communication tool for people who are speech-impaired or deaf but also plays an important role in the field of education. Lip-to-speech generation can be utilized to improve learners' speech articulation and oral expression.

However, the field of lip-to-speech generation still faces many challenges. The challenges arise from several complexities. For instance, when someone says the sound "ma" in a video (a syllable in Mandarin), different pronunciations might correspond to different meanings, like "妈" (m $\overline{a }$, referring to "mother"). These challenges in practical applications could potentially lead to decreased accuracy in lip-reading, especially when sensitive to syllabic or lexical meanings. Consequently, lip-to-speech generation in Mandarin is relatively challenging, yielding poorer recognition results and being less prevalent in this field. In summary, these challenges are multifaceted. Firstly, noise, homophones, missing context, and brief lip movement segments might result in mismatched synthesized speech with the current context, causing distortion in speech synthesis. Secondly, ensuring quality and accuracy in speech synthesis requires more efficient techniques for handling image representation quality and processing long video sequences. The broader range of lip variations in Mandarin, coupled with more homophones and synonyms, makes recognition more challenging, leading to decreased accuracy in lip-to-speech generation. These challenges make it more difficult for lip synthesis systems to match audio and visual information, posing significant obstacles to achieving accurate and natural lip-to-speech synthesis.

To solve these problems, "Lip-to-Speech Synthesis with Visual Context Attentional GAN"^2^ introduces the VCA-GAN deep architecture. This framework enables accurate speech generation by encoding images and audio, jointly modeling local and global visual features. Despite its decent performance, there's room for improvement in image representation, refinement of speech generation, and the substantial computational resources required for processing large-scale videos, leading to longer processing times. We propose the FA-GAN deep architecture, incorporating the Swin Transformer to enhance visual feature extraction. Simultaneously, a hierarchical iterative generator refines speech generation, focusing on various audio stages to boost recognition rates and generate speech closely resembling real speech. The introduction of the flash attention mechanism enhances computational efficiency, streamlining the burden and augmenting model performance. These methods have been extensively evaluated on datasets like GRID^2^ and CN-CVS^6^, yielding significant outcomes.

Collectively, The main contributions of this research include the following: (1) Introduced the FA-GAN architecture in the Chinese lip-to-speech generation domain, good recognition rates are obtained with the English datasets. (2) Implemented the Swin Transformer to optimize the existing process, enhancing the quality of image representation. (3) Introduced a hierarchical iterative generator that improved the speech generation process, enabling the model to focus more on different audio stages' features and variations, consequently improving recognition rates. 4. Introduced the flash attention mechanism to reduce the computational burden from multiple interactions of the hierarchical iterative generator, thereby enhancing the model's performance^7–10^.

## Relate work

### Lip-reading research

The origins of lip-reading research can be traced to the early 1900s, when research focused primarily on the correlation between sound production and lip and facial movements. However, as technology continued to advance, especially with the widespread use of video cameras and computers, lip-reading research gradually showed its potential. Simultaneously, lip-speaking^4^ generation techniques began to gradually evolve. In the earliest stage, lip-to-speech generation techniques relied mainly on a unimodal approach, i.e., using only a video camera to capture images and videos of the mouth and then recognizing the speech information by analysing the shape, movement, and contour of the lips. However, this approach still suffers from some challenges, such as poor image characterization and limitations such as insufficiently fine-grained speech generation processes, which limit the accuracy of lip-based speech generation.

With the rise of multimodal lip-speaking research, this means no longer relying solely on visual information and incorporating other sensors, such as microphones and sound processing techniques, to improve the accuracy of lip-speaking recognition. Yusheng Dai et al.^24^ proposed an audio-guided, cross-modal fusion encoder (CMFE) neural network that utilizes the main training parameters of multiple cross-modal attention layers to fully utilize modal complementarity. To improve audio-visual speech recognition (AVSR) within the framework of pretraining and fine-tuning training, Minsu Kim et al.^2^ proposed a lip-synthesis method based on a visual contextual attention GAN, which further improves the performance of lip recognition by better combining the visual features with the contextual context. Jeong Hun Yeo et al.^25^ proposed an audio knowledge-enabled visual speech recognition framework (AKVSR) to utilize audio modalities to supplement the shortcomings of visual modal speech information. However, these methods still face challenges in terms of speech generation and image representation. Most of these models are primarily applied to multimodal lip-reading in English. Therefore, in the Chinese context, research faces a series of challenges. In the Chinese context, there is a greater diversity in lip movements, and Chinese pronunciation and speech have unique aspects that add complexity to the model's processing.

**Figure 1:**
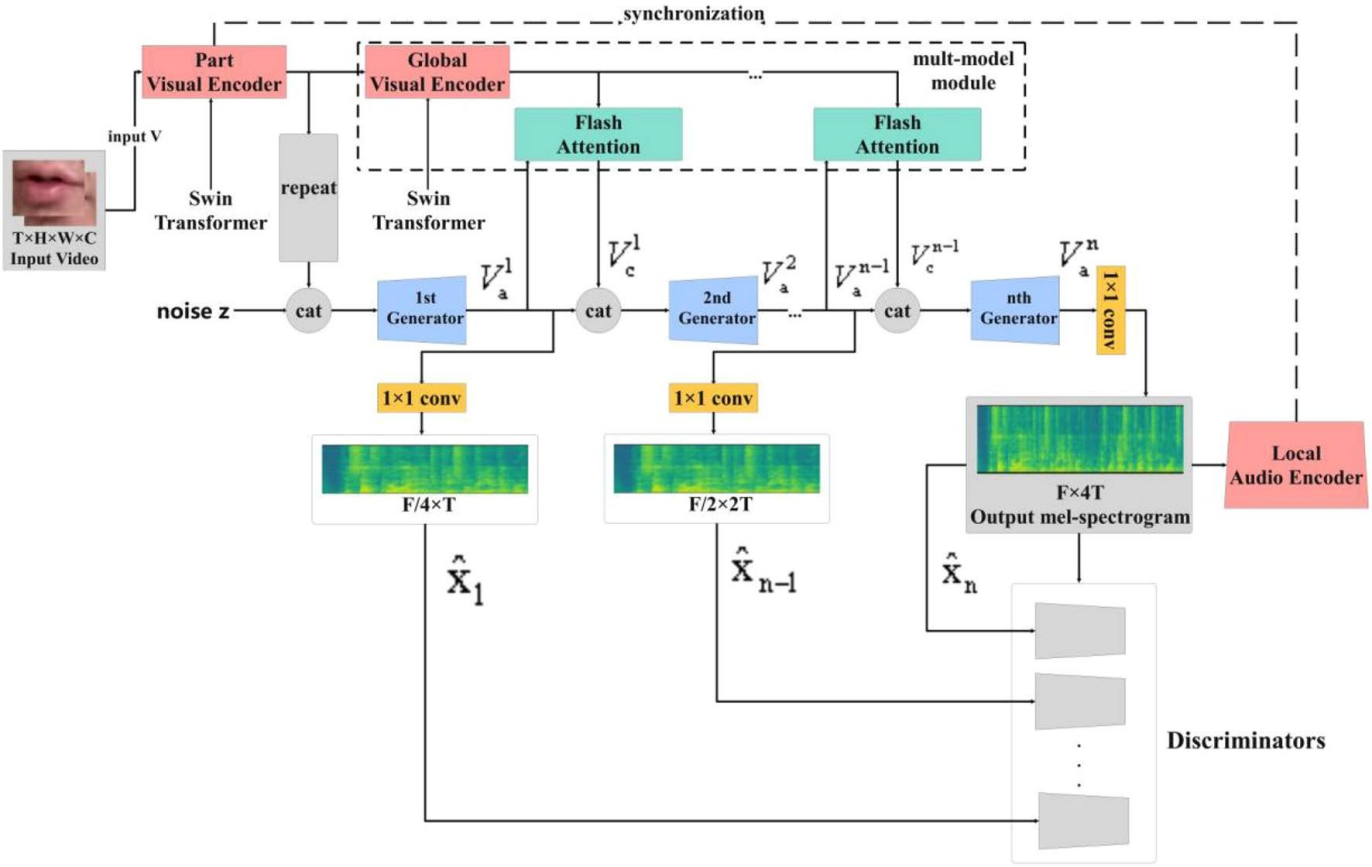
A detailed overview of the overall framework of FA-GAN is provided. Through the multimodal attention module provided in this article to the hierarchical iteration generator, flash attention is introduced to reduce the computational burden brought by the hierarchical iteration generator.

Hence, our research explicitly proposes an approach called FA-GAN, which involves separately encoding the visual and audio features of videos to fully leverage multimodal data. This approach is designed for the task of lip-to-speech generation in both Chinese and English. It aims to address issues in speech generation and image representation. The specificity of Chinese pronunciation and the diversity of lip movements in the Chinese context give this method potential advantages in the field of Chinese lip-to-speech synthesis. FA-GAN improves the extraction of local and global visual information from videos by introducing the Swin Transformer, which captures subtle variations in lip movements more accurately. Additionally, the hierarchical iterative generator is utilized to optimize the speech generation process, enabling the model to better focus on both the local and overall characteristics of speech, thereby enhancing the accuracy of the generated speech. However, this also results in a significant increase in the computational burden of the model. To mitigate these effects, the generator needs to better capture the global dependencies of the image at the middle layer through the Flash Attention module after producing speech from local features. While enhancing the accuracy of synthesized speech in both Chinese and English, lip-to-speech generation, the specific structure is illustrated in Fig. 1.

## Methods

### Proposed method

Synthesizing speech from a silent video by lip recognition is a complex task that requires precise temporal alignment of the speaker's audio speech with their lip movements and encoding of the visual features of the speaker in the video separately from the audio. Additionally, the generated speech must have the same duration as the input silent video, thus obtaining a more lifelike speech synthesis effect and improving the lip recognition accuracy. Therefore, our proposed framework involves preprocessing the input video and extracting visual features. Subsequently, in the speech generation stage, a hierarchical iterative generator is introduced to optimize and refine the speech generation. On the other hand, Flash Attention is introduced to reduce the computational burden caused by multiple interactions of the hierarchical iterative generator. The specific structure is shown in Fig. 1.

The local visual coder ${P}_{v}$ encodes the input video V into local visual features ${V}_{f}=\{{v}_{f}^{1},{v}_{f}^{2},...,{v}_{f}^{n}\}\in {\mathbb{R}}^{T\times D}$, where D is the embedding dimension. The local visual coder ${P}_{v}$ is a combination of 2D convolution and 3D convolution, and each local visual feature ${v}_{f}^{n}$ contains the edited local lip motion embedded by 3D convolution. At this point, we introduce n layers of iterative generators to generate and refine the speech representation of the obtained local visual features ${V}_{f}$ step by step from low resolution to high resolution. This process results in hierarchical audio feature generation, which allows the model to focus on the different representations and variations of the audio phase. The first generator synthesizes a coarse speech representation using the following formula: (Eq. (1))

1 $${V}_{a}^{1}={A}_{1}([\mathcal{R}({V}_{f};z)])$$

where z is noise from a standard normal distribution; $\mathcal{R}:{\mathbb{R}}^{{\text{T}}\times {\text{D}}}\to {\mathbb{R}}^{\frac{{\text{F}}}{4}\times {\text{T}}\times {\text{D}}}$ is a repetition operator that forms a 3D tensor array of height F, width T, and certain number of channels D by repeating the input features $\frac{F}{4}$ times; and [;] denotes a join or splice operation^2^.

### Detailed description

First, a video V with frame number T, height H, width W, and pass size C is provided as input. Second, after the process of feature extraction and coding, local and global visual information is captured from the video and then transformed into a feature representation. FA-GAN significantly improves speech recognition accuracy by jointly modeling local and global lip movements. This approach allows the model to better comprehend the relationship between lip movements and audio, capturing a richer visual context, thereby enhancing the quality of speech synthesis. For instance, suppose a speaker in a video has slight blurriness or local inconsistencies in lip movements. Traditional methods might struggle in recognizing specific phonemes or words due to insufficient information to determine the correct pronunciation. However, through the joint modeling of local and global lip movements in FA-GAN, the model better understands such blurry or inconsistent movements, leading to more accurate predictions of the correct pronunciation. For example, a speaker utters a phoneme "ba" in a video. During a certain period of local lip movement, there might be some blurriness or inconsistency due to video quality or other reasons, which traditional methods could inaccurately recognize. However, FA-GAN integrates local blurry lip movements with the overall context, comprehending the specific phoneme the speaker is pronouncing more comprehensively, hence generating the corresponding speech more accurately. This joint modeling approach enables the model to handle local noise or blurriness better, grasping the speaker's intention holistically, thereby enhancing speech recognition accuracy. Third, an attention mechanism is used to align the information from audio and video. By learning the weights, the attention mechanism can identify the appropriate alignment to ensure that the synthesized speech matches the video. The generated speech is ${\text{S}}\in {\mathbb{R}}^{{\text{M}}\times 4{\text{T}}}$, where M denotes the dimension of the Meier spectrum and 4T denotes that the frame length of the speech is four times that of the video. This outcome is achieved by applying a short-time Fourier transform and frame shift operation to ensure that the speech duration matches the video. To further improve the quality of speech synthesis, we introduce a hierarchical iterative generator that assigns the task of synthesizing speech to n generators, which further refines the speech synthesis process so that the model can focus on different stages of audio changes and improve the accuracy of speech synthesis.

To reduce the computational burden of the hierarchical iterative generator, we introduce the flash attention mechanism. This mechanism automatically learns the weights of each modality (audio and image) to facilitate better information transfer and interaction between them. This approach not only helps model the temporal relationship between audio and images but also improves the performance of the model and reduces the computational burden. Notably, we treat the Mel spectrogram as an image and use a 2D GAN to train the model. The advantage of this approach is that it efficiently handles the alignment and fusion between audio and video to generate more realistic speech and improves the performance of the model^2^.

### Flash attention GAN

When considering lip-to-speech synthesis from silent video input, the emphasis is on focusing on local lip movements while also considering the overall context of the video. This not only provides additional information but also reduces interference from homophones based on the tone. Therefore, our research explicitly proposes an approach called FA-GAN, which involves modeling and training on both local visual features and global visual context, fully leveraging multimodal data. The introduction of the Swin Transformer enhances the model's capacity to extract both local and global visual information from videos, enabling a more precise capture of subtle variations and features in lip movements. This enhancement provides the model with superior image representation capabilities, enabling a more accurate interpretation and synthesis of lip movements and speech.

Additionally, to improve the speech generation process, we employ a hierarchical iterative generator. This approach enables the model to better focus on the local and overall characteristics of speech, thereby enhancing the accuracy of the generated speech. This approach allows the model to improve and enhance the synthesis process at various levels, targeting different stages to enhance speech features and representations, ultimately enhancing the quality and authenticity of the generated speech. However, this also results in a significant increase in the computational burden of the model. To mitigate these effects, the generator needs to improve its capture of the global dependencies of the image at the middle layer through the Flash Attention module after producing speech from local features. By applying the attention mechanism into the intermediate layers of the generator, the model can focus more effectively on local features and global dependencies. As a result, during the speech generation process, the model can effectively focus on important parts of the image instead of globally processing the entire image. This refined approach significantly reduces the use of computational resources and improves computational efficiency. The specific structure is shown in Fig. 1.

### Swin Transformer

**Figure 2:**
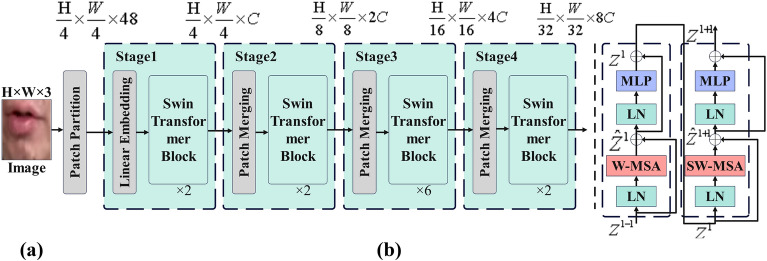
(**a**) Architectural diagram depicting the principles of the Swin Transformer utilized in the framework. (**b**) Two successive Swin-transformer blocks: window-based multihead self-attention (W-MSA) and sliding window multi-head self-attention (SW-MSA).

The Swin Transformer is a deep learning model based on the Transformer architecture, designed for computer vision tasks such as image classification, object detection, and semantic segmentation. The specific framework diagram is shown in Fig. 2. It divides the input image into non-overlapping image blocks called "windows." These windows compose a "window partition." Within each window, the model performs self-attention operations to capture local features within the image. Through a cascade of window partitions and self-attention operations, the model gradually acquires features from different levels of the image, achieving hierarchical context. To reduce the computational complexity, the Swin Transformer introduces the shifted window strategy, which restricts self-attention operations to take place within a specific window rather than calculating globally. This window can shift across different resolution levels, thus covering the entire image. Furthermore, to maintain the continuity of image features, the Swin Transformer introduces cross-window connections^5^.

The introduction of the Swin Transformer has had a significant impact on the image representation within FA-GAN. By enhancing the extraction of local and global visual information from the video, it has empowered the model with improved image representation capabilities. This enhancement is crucial for accurate lip-to-speech synthesis as it enables more precise capture of subtle variations and features in lip movements, allowing the model to precisely comprehend and synthesize mouth shapes and pronunciations. Lip-to-speech synthesis necessitates the model's ability to accurately capture details of lip movements and features across different stages to generate synthesized speech closer to real human speech. The Swin Transformer, by better capturing both local and global information within the video, has provided the model with more expressive and accurate image representations, significantly contributing to the creation of more natural and lifelike speech synthesis effects.

### Attention mechanisms

Have had a significant impact in various research areas, such as facial recognition, machine translation, lip-reading, and speech recognition. Their main advantage lies in their ability to focus on relevant information, effectively reducing interference from irrelevant information. Furthermore, attention mechanisms have begun to be applied in fields such as generative adversarial networks (GANs). For instance, research teams led by Chan and Jaitly^22^ introduced attention mechanisms to convert the alignment problem between acoustic features and text sequences into a sequence-to-sequence problem, leading to significant performance improvements in speech recognition tasks. In this study, the hierarchical iterative generator introduced improves the quality of coarse speech by jointly modeling local and global visual features, leading to hierarchical audio feature generation. This allows the model to focus on different representations and variations of the audio phase. However, it also results in an increase in computational load because of the multiple interactions with the hierarchical iterative generator during the speech synthesis process. Therefore, we propose a multimodal attention module that consists of a global visual encoder and flash attention. This aims to decrease the significant computational load caused by multiple interactions with the hierarchical iterative generator during the speech synthesis process. The basic principle is as follows: the global visual coder ${G}_{v}$ generates the global visual feature ${C}_{v}\in {\mathbb{R}}^{T\times D}$ by considering the relationships among the whole local visual features ${V}_{f}$ through the Swin Transformer network. Flash attention then proceeds to look for complementary cues related to speech synthesis, considering the importance of the global visual feature ${C}_{v}$ in relation to the i-th resolution speech representation ${V}_{a}^{i}\in {\mathbb{R}}^{{V}_{i}\times {T}_{i}\times {D}_{i}}$ Simultaneously, flash attention simplifies the computational steps for long sequences and reduces the read/write operations to HBM/SRAM, which is mainly based on the following formulas:

2 $${B}_{c}=[\frac{M}{4d}],{B}_{r}=min([\frac{M}{4d}],d),{T}_{r}=[\frac{N}{{B}_{r}}],{T}_{c}=[\frac{N}{{B}_{c}}]$$

3 $${Q}_{i}=\frac{Q}{{T}_{r}},{K}_{i}=\frac{K}{{T}_{c}},{V}_{i}=\frac{V}{{T}_{c}}$$

4 $${V}_{c}^{i}=S(softmax(\frac{{Q}_{i}{K}_{i}^{T}}{\sqrt{d}}){V}_{i})$$

**Figure 3:**
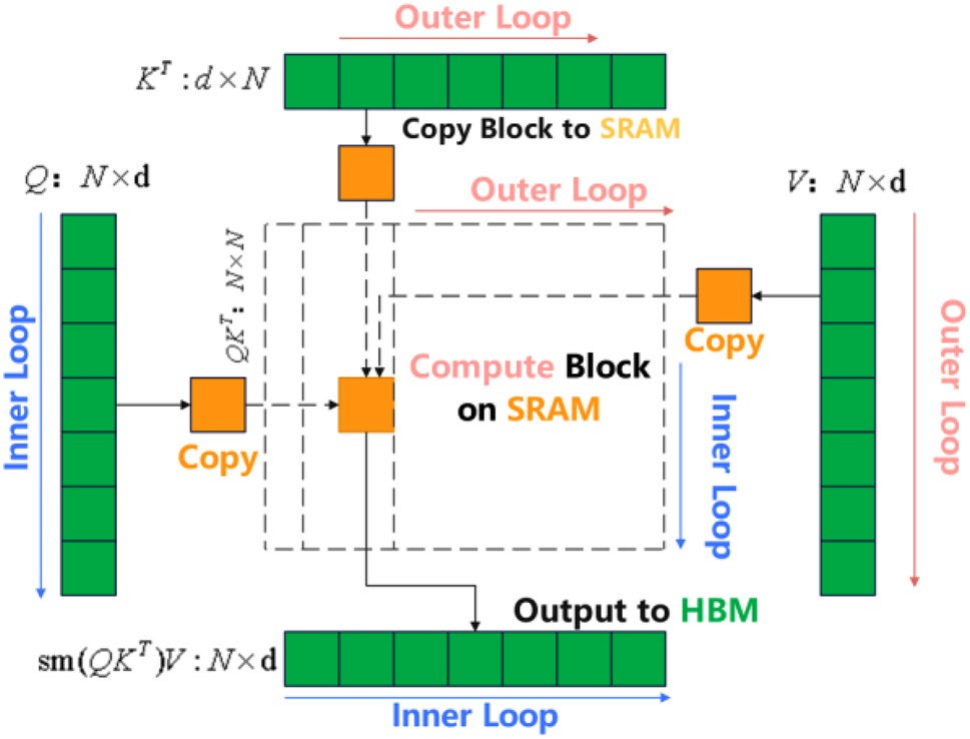
Overview and modelling of the flash attention principle proposed in this article.

Figure 3 is the operational flowchart of Flash attention. Based on the size of the SRAM, the size of the row/column modules is calculated. Then, the output matrix ${\text{O}}=(0{)}_{{\text{N}}\times {\text{d}}}\in {\mathbb{R}}^{{\text{N}}\times {\text{d}}}$ is initialized with all zeros, which will serve as an accumulator. The purpose of $\uptau =(0{)}_{{\text{N}}}\in {\mathbb{R}}^{{\text{N}}}$ is to store the cumulative denominator of the softmax operation (i.e., the sum of exp scores). ${\text{M}}=(-\infty {)}_{{\text{N}}}\in {\mathbb{R}}^{{\text{N}}}$ is used to store the maximum score row-wise. Next, per the specifications of Eq. (2), Q, K, and V are partitioned into blocks of the same size. Similarly, O, $\uptau$, and M are partitioned with the same block size. Next, a cross-column loop is initiated, which means traversing key/value vectors. ${{\text{K}}}_{{\text{i}}}$ and ${{\text{V}}}_{{\text{j}}}$ blocks are loaded from HBM to SRAM. However, at this point, 50% of SRAM remains unused, specifically reserved for Q and O. Consequently, it enters an inner cross-row loop, which involves traversing query vectors. ${{\text{Q}}}_{{\text{i}}}$ and ${{\text{O}}}_{{\text{i}}}$ blocks and ${\uptau }_{{\text{i}}}$ and ${{\text{M}}}_{{\text{i}}}$ are loaded into SRAM. The dot product between ${{\text{Q}}}_{{\text{i}}}({{\text{B}}}_{{\text{r}}}\times {\text{d}})$ and the transposed ${{\text{K}}}_{{\text{j}}}^{{\text{T}}}$ is computed according to Eq. (5), resulting in ${{\text{S}}}_{{\text{ij}}}$.

5 $${S}_{ij}={Q}_{i}{K}_{j}^{T}\in {\mathbb{R}}^{{B}_{r}\times {B}_{c}}$$

Next, the scores calculated in the previous step are utilized to calculate $\widetilde{{{\text{M}}}_{{\text{ij}}}}$, $\widetilde{{\uptau }_{{\text{ij}}}}$, and $\widetilde{{{\text{P}}}_{{\text{ij}}}}$ following Eqs. (6 and 7), and the row-wise sum of the matrix P is obtained per the equation. Then, in accordance with Eq. (8), ${{\text{M}}}_{{\text{i}}}^{{\text{new}}}$ and ${\uptau }_{{\text{i}}}^{{\text{new}}}$ are computed. Subsequently, according to the definition in Eq. (9), the matrix ${{\text{O}}}_{{\text{i}}}$ (where the exp term is used to modify $\widetilde{{{\text{P}}}_{{\text{ij}}}}$ and ${{\text{O}}}_{{\text{i}}}$ to eliminate the effect of the previous iteration's M) is derived^9^. As specified in Eq. (10).

6 $$\widetilde{{{\text{M}}}_{{\text{ij}}}}={\text{rowmax}}({{\text{S}}}_{{\text{ij}}})\in {\mathbb{R}}^{{{\text{B}}}_{{\text{r}}}},\widetilde{{{\text{P}}}_{{\text{ij}}}}={\text{exp}}({{\text{S}}}_{{\text{ij}}}-\widetilde{{{\text{M}}}_{{\text{ij}}}})\in {\mathbb{R}}^{{{\text{B}}}_{{\text{r}}}\times {{\text{B}}}_{{\text{c}}}}({\text{pointwise}})$$

7 $$\widetilde{{\uptau }_{{\text{ij}}}}={\text{rowsum}}(\widetilde{{{\text{P}}}_{{\text{ij}}}})\in {\mathbb{R}}^{{{\text{B}}}_{{\text{r}}}}$$

8 $${{\text{M}}}_{{\text{i}}}^{{\text{new}}}={\text{max}}({{\text{M}}}_{{\text{i}}},\widetilde{{{\text{M}}}_{{\text{ij}}}})\in {\mathbb{R}}^{{{\text{B}}}_{{\text{r}}}},{\uptau }_{{\text{i}}}^{{\text{new}}}={{\text{e}}}^{{{\text{M}}}_{{\text{i}}}-{{\text{M}}}_{{\text{i}}}^{{\text{new}}}}{\uptau }_{{\text{i}}}+{{\text{e}}}^{\widetilde{{{\text{M}}}_{{\text{ij}}}}-{{\text{M}}}_{{\text{i}}}^{{\text{new}}}}\widetilde{{\uptau }_{{\text{ij}}}}\in {\mathbb{R}}^{{{\text{B}}}_{{\text{r}}}}$$

9 $${{\text{O}}}_{{\text{i}}}\leftarrow {\text{diag}}({\uptau }_{{\text{i}}}^{{\text{new}}}{)}^{-1}({\text{diag}}({\uptau }_{{\text{i}}}){{\text{e}}}^{{{\text{M}}}_{{\text{i}}}-{{\text{M}}}_{{\text{i}}}^{{\text{new}}}}{{\text{O}}}_{{\text{i}}}+{{\text{e}}}^{\widetilde{{{\text{M}}}_{{\text{ij}}}}-{{\text{M}}}_{{\text{i}}}^{{\text{new}}}}\widetilde{{{\text{P}}}_{{\text{ij}}}} {{\text{V}}}_{{\text{j}}})$$

10 $${\uptau }_{{\text{i}}}\leftarrow {\uptau }_{{\text{i}}}^{{\text{new}}},{{\text{M}}}_{{\text{i}}}\leftarrow {{\text{M}}}_{{\text{i}}}^{{\text{new}}}$$

The obtained global visual joint feature ${V}_{c}^{i}$ is connected to the unrefined speech ${V}_{a}^{i}$, which is subsequently and iteratively improved in a hierarchical iterative generator using the following formula (Eq. (11)):

11 $${V}_{a}^{i+1}={A}_{i}([{V}_{a}^{i};{V}_{c}^{i}]),for i=\mathrm{1,2},...,n-1$$

Thus, the following generator allows us to obtain cues for synthesizing accurate speech from the global visual environment in the process of obtaining phoneme mappings using local visual features. Additionally, to generate more fine-grained images (e.g., Mel spectrograms), we use multiple discriminators^2^. From the generated multiscale Mel spectrogram $(\widehat{{{\text{x}}}_{1}},\widehat{{{\text{x}}}_{2}},...,\widehat{{{\text{x}}}_{{\text{n}}}})$, each generated speech representation ${(V}_{a}^{1},{V}_{a}^{2},...,{V}_{a}^{n})$ after 1*1 convolution is passed to the multiple discriminator, as shown in Fig. 1.

### Synchronization^2^

Since humans are very sensitive to audio-visual synchronization, it is important to synthesize the input lip-synchronized video with synchronized speech. To generate synchronized speech, we use two strategies: 1. Speech is synthesized by maintaining the temporal representation of the input video. Our approach ensures that the generated speech is temporally consistent with the video to ensure audiovisual synchronization. The generator is directed to focus on synchronization. As previously mentioned, each visual representation ${v}_{f}^{n}$ encoded by ${P}_{v}$ contains lip motion information at the local level. Our goal in designing the generator is to synthesize speech based on local visual features ${V}_{f}$ without destroying this information. In this way, the output speech can be naturally synchronized by phonemic-to-phoneme mapping without disturbing its temporal information. Furthermore, to ensure synchronization, we employ the modern concept of deep synchronization, which learns in a self-supervised manner not only audio-visual synchronization representations but also discriminative representations. To achieve this goal, a local audio encoder ${{\text{LOC}}}_{{\text{a}}}$, which encodes local audio features ${{\text{V}}}_{{\text{a}}}=\{{{\text{v}}}_{{\text{a}}}^{1},{{\text{v}}}_{{\text{a}}}^{2},...,{{\text{v}}}_{{\text{a}}}^{{\text{n}}}\}\in {\mathbb{R}}^{{\text{T}}*{\text{D}}}$ from the ground truth Mel spectrogram Q, is introduced. Using the encoded local audio features ${{\text{V}}}_{{\text{a}}}$ and local visual features ${V}_{f}$, we perform a comparative learning of the synchronization representation by using the following InfoNCE loss^21^ (Eq. (12)):

12 $${E}_{c}({V}_{a},{V}_{f})=-{\mathbb{E}}[log(\frac{exp(cos({v}_{a}^{j},{v}_{f}^{j})/\tau )}{{\sum }_{n}exp(cos({v}_{a}^{j},{v}_{f}^{n})/\tau )})]$$

where cos is the cosine similarity measure, τ is the temperature parameter used to control the scale or smoothness of the probability distribution in some deep learning models, and ${V}_{a}$ and ${V}_{f}$ have the same time horizon. The loss function serves to guide the allocation of high similarity to pairs that are aligned between audio representations and visual representations while allocating low similarity to pairs that are not aligned. We obtain the synchronization loss (Eq. (13)) for both encoders:

13 $${{\text{E}}}_{{\text{e}}\_{\text{sync}}}=\frac{1}{2}({{\text{E}}}_{{\text{c}}}({{\text{V}}}_{{\text{a}}},{{\text{V}}}_{{\text{f}}})+{{\text{E}}}_{{\text{c}}}({{\text{V}}}_{{\text{f}}},{{\text{V}}}_{{\text{a}}}))$$

where the second term is formed by applying a symmetric approach to the negative audio samples, similar to Eq. (12). In addition, the audio feature $\widehat{{V}_{a}^{n}}$ encoded from the final generated Mayer spectrogram $\widehat{{{\text{x}}}_{{\text{n}}}}$ is compared with the visual representation to guide the generator in synthesizing synchronized speech. This comparison is performed by means of the loss function (Eq. (14)):

14 $${{\text{E}}}_{{\text{g}}\_{\text{sync}}}=||1-{\text{cos}}(\widehat{{V}_{a}^{n}} ,{{\text{V}}}_{{\text{f}}})|{|}_{1}$$

which maximizes the cosine similarity between the generated audio features and the given visual features, thus synchronizing the generated Mel spectrogram with the input video. The final loss of synchronization is defined as:

15 $${E}_{sync}={{\text{E}}}_{{\text{e}}\_{\text{sync}}}+{{\text{E}}}_{{\text{g}}\_{\text{sync}}}$$

### Waveform conversion^2^

The Mel spectrograms generated in this study have a wide range of applications in several speech-related tasks, including automatic speech recognition and audiovisual speech recognition. However, to improve the accuracy of speech recognition and to produce an auditory experience that resembles real speech, we also need to convert the generated Mel spectrograms into sound waveforms. To accomplish this conversion task, existing vocoder algorithms are usually employed. In our study, on the other hand, we chose to use the Griffin-Lim^17^ algorithm, which is a classical audio signal processing method that is mainly utilized to reduce linear spectrograms to their corresponding audio waveforms. The main application of this algorithm is to perform audio signal inversion, i.e., to reduce the sound signal from spectral information. However, in this study, our model generates Mel spectrograms instead of linear spectrograms. Therefore, before using the Griffin-Lim algorithm, we need to process the model through an additional postprocessing network, PostNet, which is a neural network architecture specifically designed to improve the quality of the generated spectrograms. PostNet further processes the spectrograms through operations such as convolution, residual joining, and batch normalization to improve the quality of the synthesized speech and the audibility. Therefore, we chose to use PostNet in this session to map the generated Mel spectrograms to linear spectrograms. While training PostNet, we used the L1 reconstruction loss function and compared the generated results with the real linear spectrogram to ensure the accuracy of the conversion process. These training strategies help generate high-quality sound waveforms that match the Mel spectrogram, providing high-quality speech data for subsequent speech synthesis and recognition tasks. This approach allows us to establish meaningful mapping relationships that allow the generated sounds to perform well in a variety of speech applications^2^.

### Loss function^2^

To make the final generated Meier spectrograms more realistic, we introduce a loss function while defining the objective function of the FA-GAN generator part as (Eq. (16)):

16 $${\text{E}}={{\text{E}}}_{{\text{GAN}}}+{\uplambda }_{{\text{recon}}}{{\text{E}}}_{{\text{recon}}}+{\uplambda }_{{\text{sync}}}{{\text{E}}}_{{\text{sync}}}$$

where ${\uplambda }_{{\text{recon}}}$ is the weight parameter used for reconstruction loss and ${\uplambda }_{{\text{sync}}}$ is the weight parameter used for synchronization loss. ${{\text{E}}}_{{\text{GAN}}}$ denotes the generated loss, ${{\text{E}}}_{{\text{recon}}}$ is the reconstruction loss, and ${{\text{E}}}_{{\text{sync}}}$ is the synchronization loss, which collectively simulate the conditional and unconditional distributions as follows (Eq. (17)):

17 $${{\text{E}}}_{{\text{GAN}}}=-\frac{1}{2}{\mathbb{E}}_{{\text{i}}}[{{\text{logD}}}_{{\text{i}}}(\widehat{{\text{x}}})+{{\text{logD}}}_{{\text{i}}}(\widehat{{{\text{x}}}_{{\text{i}}},}{\text{M}}({{\text{C}}}_{{\text{v}}}))]$$

where ${{\text{D}}}_{{\text{i}}}$ denotes the ith defender. The first half denotes the unconditional GAN loss term, which is used to ensure that the generated Mel spectrogram is similar to the real Mel spectrogram to improve the fidelity of the generated image. The second half represents the conditional GAN loss term, which is used to bootstrap the generated Mel spectrogram to match the global visual context M(Cv), where M(-) represents the time-averaged pooling operation. This process ensures that the generated images are consistent with the global visual information. To complete the GAN training, the discriminator loss is defined as (Eq. (18)):

18 $$E_{d} = - \frac{1}{2}{\mathbb{E}}_{i} \left[ {logD_{i} \left( {x_{i} } \right) + log\left( {1 - D_{i} \left( {\hat{x}_{i} } \right)} \right) + logD_{i} \left( {x_{i} ,M\left( {C_{v} } \right)} \right) + log\left( {1 - { }D\left( {\hat{x}_{i} ,M\left( {C_{v} } \right)} \right)} \right)} \right]$$

The reconstruction loss ${E}_{recon}$ is defined in terms of the ${L}_{1}$ distance between the generated truth spectrogram at resolution i and the ground truth spectrogram^2^ (Eq. (19)):

19 $${E}_{recon}={\mathbb{E}}_{i}[||{x}_{i}-{\widehat{x}}_{i}|{|}_{1}]$$

## Experiment and results

### Experimental preparation

CN-CVS^6^ is currently the largest and most populous, multimodal Chinese dataset that is available, with more than 2500 speakers, more than 200,000 data entries, and a total duration exceeding 300 h. The dataset is primarily divided into two sections—News and Speech—with data sourced from television news programs and speech-oriented web content.In this paper, only single-speaker data are used for correction, and the model is evaluated under four different environmental conditions.

The GRID^2^ dataset comprises video and audio recordings from hundreds of speakers, showcasing their diverse mouth shapes and speech patterns used to articulate words and phrases. These meticulously recorded and annotated data aim to explore the correlation between speech and mouth movements. Each speaker's video data includes lip movements, recorded on a word or phrase basis using multiple cameras to capture various perspectives of lip movements. The GRID dataset offers comprehensive multimodal annotations, encompassing lip positions in the video and speech transcripts in the audio.

### Implementation details^2^

For the visual encoder part, we adopted the Swin Transformer architecture, which is currently one of the most popular architectures in the field of computer vision. When the input image reaches the Swin Transformer architecture, it undergoes several steps. Patch Partition Module: the image is divided into small patches and flattened in the channel direction. Linear Embedding Layer: a linear transformation is applied to the channel data of each pixel to alter the feature representation. Swin Transformer Block: feature maps are constructed through four different stages, and Swin Transformer blocks are stacked to extract visual features. Layer normalization is applied to normalize the features. A global pooling operation is performed to reduce the dimension of the features. The output is obtained through a fully connected layer. The audio encoder part includes two convolutional layers with a stride of 2 and a residual block to extract audio features. PostNet^2^ consists of three 1D residual blocks and two 1D convolutional layers to improve the quality of the generated spectrogram. The discriminator typically consists of 2, 3, or 4 residual blocks to determine whether the generated spectrogram is real. In terms of data preprocessing, the audio is resampled to 16 kHz, is high-pass filtered, and is then converted to a Mel spectrogram with 80 Mel filter banks (F = 80). For video datasets with different frame rates, different window sizes and hop sizes are used to obtain matching Mel spectrograms. During training, the random sampling sizes for consecutive sequences are 40 and 50, while during inference, the network can handle video frames of any length to generate speech. For multiscale ground truth Mel spectrograms (${x}_{1}$ and ${x}_{2}$), bilinear interpolation is applied to the ground truth Mel spectrogram ${x}_{3}$. The optimizer uses Adam with a learning rate of 0.0001. α, ${\lambda }_{recon}$, and ${\lambda }_{sync}$ are set to 2, 50, and 0.5, respectively, and the temperature parameter τ is set to 1. For GAN loss, nonsaturating adversarial loss and R1 regularization are employed. The computations are performed using an NVIDIA 3090.

### Hyper-parameter comparative experiment

*Hyper-parameters* Considering the selection of hyper-parameters, we've introduced Extended Short-Time Objective Intelligibility (ESTOI) as a criterion to gauge the suitability of these parameters for the model. The hyper-parameters included in this paper are: "${L}_{r}$(learning_rate)", "α" adjusts the weights or influence of different parts within the loss function. "T(Temperature parameter)". Along with the loss function's ${\lambda }_{recon}$ and ${\lambda }_{sync}$.

This dissertation conducted comparative experiments on different parameters using an 2080ti GPU on the GRID dataset. The “${L}_{r}$(learning_rate)” was set between 0.0001 and 0.1, “α” ranged from 1 to 3, and the temperature parameter “T” varied between 0.05 and 1. Various numerical experiments were conducted on “α”, “ λ”, “T”, and “ ${L}_{r}$”. According to the results in Table 1, when ${L}_{r}$= 0.0001, α = 2, ${\lambda }_{recon}$= 50, and ${\lambda }_{sync}$= 0.5, the model exhibited better performance, whereas variations in the temperature parameter “T” had minimal impact on the results. Therefore, the choice of these parameters significantly affects the model's performance.

**Table 1:** The table primarily tests the impact of all hyper-parameters in this paper on the model's performance.

Parameters	Number of parameters	ESTOI
${L}_{r}$(learning_rate)	0.01	0.514
	0.001	0.607
	**0.0001**	**0.625**
α	1	0.624
	**2**	**0.629**
	3	0.618
T	**1**	**0.629**
	**0.05**	**0.629**
${\lambda }_{recon}$	1	0.413
	20	0.574
	**50**	**0.629**
${\lambda }_{sync}$	1	0.577
	**0.5**	**0.629**
	0.2	0.615

Bold values represent the optimal values.

### Comparative experiment

#### Experiment description

For our comparative experiments, we introduced five evaluation metrics: Short-TimeObjective Intelligibility (STOI), Extended Short-Time Objecti-ve Intelligi-

Bility (ESTOI), Perceptual Evaluation of Speech Quality (PESQ), word error rate (WER) and Mean Option Scores (MOS).

These metrics collectively help us assess the performance of the proposed method on different datasets. According to the results in Table 2, we can see that our designed framework performs exceptionally well on the English dataset GRID, especially outperforming existing deep learning architectures in terms of ESTOI and achieving excellent recognition rates.However, due to the complexity of the Chinese language, progress in the field of Chinese lip-reading recognition has been relatively slow. Chinese includes numerous characters and phonemes, making its pronunciation more intricate. The presence of homophones and tones further increases the difficulty of recognition. Based on the results in Table 3, we observe that our designed framework excels on the Chinese dataset CN-CVS, with all metrics surpassing existing architectures. A particularly high level of recognition accuracy is achieved in this field.

**Table 2:** Performance of the FA-GAN framework proposed in this article on GRID.

Method	STOI	ESTOI	PESQ	WER (%)	MOS
Vid2Speech^11^	0.491	0.335	1.734	44.92	2.8
Lip2AudSpec^12^	0.513	0.352	1.673	32.51	3.4
1D GAN-based^13^	0.564	0.361	1.684	26.64	3.5
Lip2Wav^14^	**0.731**	0.535	1.772	14.08	4.3
VAE-based^15^	0.724	0.540	1.932	–	4.2
Vocoder-based^16^	0.648	0.455	1.900	23.33	3.9
VCA-GAN^2^	0.724	0.609	**2.008**	**12.25**	**4.6**
**FA-GAN**	0.724	**0.625**	1.939	12.67	**4.6**

Bold values represent the optimal values.

**Table 3:** Performance of the FA-GAN framework proposed in this article on CN-CVS.

Method	STOI	ESTOI	PESQ	WER (%)	MOS
GAN-based^13^	0.414	0.306	1.596	63.57	2.6
VAE-based^15^	0.528	0.412	1.623	52.71	3.2
VCA-GAN^2^	0.598	0.471	1.604	49.70	3.4
**FA-GAN**	**0.614**	**0.580**	**1.772**	**43.19**	**4.0**

Bold values represent the optimal values.

### Ablation experiment

To validate the functionality of each module in FA-GAN, we conducted ablation experiments using the CN-CVS dataset. The results are shown in Table 4.We progressively introduced each module, constructing four different model configurations, as shown in the table above. To measure WER, we utilized a pretrained ASR model on the CN-CVS dataset under the same settings. First, we removed the Swin-Transformer module from the original design. In this configuration, STOI was 0.528, ESTOI was 0.412, PESQ was 1.623, MOS was 3.4 and WER was 52.17%. All metrics experienced a decrease. Second, we retained the Swin-Transformer module but removed the Multi Generator module. In this scenario, STOI was 0.439, ESTOI was 0.392, PESQ was 1.608, MOS was 2.8 and WER was 58.03%. The metrics exhibited a more significant decline, which suggests that the hierarchical iterative generator plays a critical role in speech generation. The generator not only refines the speech generation process but also significantly influences the accuracy of the generated speech. Subsequently, we retained the Swin-Transformer module and Multi Generator module but replaced flash attention module with cross-attention^28^. In this configuration, STOI was 0.574, ESTOI was 0.500, PESQ was 1.683, MOS was 3.9 and WER was 48.69%. Last, when we retained all modules, the STOI was 0.614, the ESTOI was 0.580, the PESQ was 1.772, the MOS was 4.0 and the WER was 43.19%. This finding indicates that these three modules play a crucial role in the model, and the resulting WER of 43.19% is the lowest error rate in Chinese lip-reading to speech generation.

**Table 4:** Ablation experiments of different modules in the FA-GAN framework on CN-CVS.

Modules				Efficiency				
Base-line	Swin-transformer	Flash attention	Multi generator	STOI	ESTOI	PESQ	WER (%)	MOS
√	×	√	√	0.528	0.412	1.623	52.71	3.4
√	√	√	×	0.439	0.392	1.608	58.03	2.8
√	√	×	√	0.574	0.500	1.683	48.69	3.9
√	√	√	√	**0.614**	**0.580**	**1.772**	**43.19**	**4.0**

Bold values represent the optimal values.

### Inference time experiment

*Inference Time* refers to the time needed for a deep learning model to process input data and generate output results. We conducted performance tests on both the original framework and our improved framework using the same CN-CVS dataset and the identical NVIDIA 3090 graphics card. As indicated in Table 5, our improved framework outperforms the original framework in terms of "Inference time," "ESTOI," and "Word Error Rate."

**Table 5:** Comparison of performance between the original framework and the improved framework in this article on CN-CVS.

Method	Inference time (ms)	ESTOI	WER (%)
VCA-GAN^2^	23.67	0.471	49.70
FA-GAN	18.24	0.580	43.19

### Performance experiment of hierarchical iterative generator

Learning visual features in videos may simultaneously have a negative impact not only on model performance and but also the recognition rate. By introducing hierarchical iteration, we can gradually guide the model to learn from shallow layers to deep layers, reduce the burden on the model, and improve the recognition rate. The results in Table 6 show that different iteration levels will lead to a continuous increase in ESTOI, but after a certain stage, ESTOI will tend to stabilize.

**Table 6:** Output performance of hierarchical iteration generators in different layers.

Number of layers	ESTOI
1	0.3
2	0.485
3	0.580
4	0.510
5	0.498
6	0.500
…	…
10	0.500

### Results analysis and comparisons

Based on all the results above, our study demonstrates that the performance of the proposed FA-GAN framework on the English dataset GRID indicates a slight lag in the STOI metric compared to the Lip2Wav14^14^ model, with a decrease of 0.007. However, in terms of the ESTOI metric, the FA-GAN model outperforms similar models by achieving the highest level. In contrast, in terms of the PESQ and WER metrics, it slightly lags behind the VCA-GAN model, with a decrease of 0.069 and 0.42%, respectively. On the Chinese dataset CN-CVS, the FA-GAN model outperforms the comparable models in all metrics, particularly the WER metric, achieving a significantly lower rate of 43.19% compared to other models.

The uniqueness of the FA-GAN model lies in its complete utilization of multimodal data. Compared to other models, it can comprehensively integrate visual and audio information, thereby enhancing the accuracy of lip-to-speech synthesis. The introduction of the Swin Transformer significantly enhances the model's capacity to extract both local and global visual information from videos. This advantage allows the model to more accurately capture subtle variations in lip movements, enhancing the quality of image representation. The FA-GAN utilizes a hierarchical iterative generator to optimize the speech generation process, enabling the model to better focus on the local and overall characteristics of speech, thereby enhancing the accuracy of the generated speech. In the Chinese domain, the FA-GAN model still encounters several challenges, such as the increased diversity in lip movements and the inherent complexity of Chinese pronunciation and speech. In the field of lip-to-speech synthesis, FA-GAN stands out for its unique and important contributions, including the innovative integration of multimodal data, the introduction of an advanced Transformer structure, and the optimization of the speech generation process. Its potential benefits in Chinese lip-to-speech synthesis position it as a significant contribution to relevant research.

## Discussion

After analyzing the model we proposed, it lags slightly behind other existing English sentence-level lip-to-speech synthesis frameworks in metrics such as WER, STOI, PESQ, etc. Therefore, there are still numerous challenges ahead, and we will continually refine the model in our future work to achieve higher levels of performance. Future research extensions may include: (a) improving attention accuracy and efficiency for better feature capturing; (b) considering the incorporation of facial expressions to optimize speech emotions and enhance overall model performance; (c) further researching methods to enhance the model's adaptability to various noises and environmental changes, as well as handling variations in lip movements and pronunciations, to ensure robust performance in different scenarios. Additionally, exploring the model's application in multilingual environments could expand its practical utility. For the advancement of lip-to-speech generation technology, we should also explore the integration of more advanced deep learning and natural language processing techniques to enhance the overall performance and applicability of the model. These research directions will contribute to further advancements in the field of lip-to-speech synthesis and offer more comprehensive solutions for practical application scenarios.

## Conclusion

In this study, we have introduced a new sentence-level lip-to-speech synthesis architecture, FA-GAN, for the first time in the Chinese lip-to-speech synthesis domain. The aim is to tackle issues related to visual ambiguity, inadequate extraction of image features, and insufficient refinement in the speech generation process. To address the issue of inadequate image feature extraction, we utilized the Swin Transformer to improve the extraction of both local and global visual information from videos. By implementing an attention mechanism, we have minimized visual ambiguity, reduced computational load, and ensured that the synthesized speech aligns with the video. Additionally, we have implemented a hierarchical iterative generator to improve the accuracy and naturalness of speech generation. Experimental results on the GRID and CN-CVS datasets demonstrated that the proposed FA-GAN architecture outperforms existing Chinese Mandarin sentence-level lip-to-speech synthesis frameworks in metrics such as STOI and ESTOI. It also outperforms current English sentence-level lip-to-speech synthesis frameworks in metrics such as ESOI and MOS.

## Data Availability

Both CN-CVS and GRID are publicly available and free datasets without certification.
